# Early SARS-CoV-2 infection: Platelet-neutrophil complexes and platelet function

**DOI:** 10.1016/j.rpth.2022.100025

**Published:** 2022-12-23

**Authors:** Marina Rieder, Niklas Baldus, Daniela Stallmann, Maren Jeserich, Isabella Goller, Luisa Wirth, Luisa Pollmeier, Maike Hofmann, Christoph Bode, Hans-Joerg Busch, Bonaventura Schmid, Nadine Gauchel, Rüdiger E. Scharf, Daniel Duerschmied, Achim Lother, Krystin Krauel

**Affiliations:** 1 Interdisciplinary Medical Intensive Care, Medical Center – University of Freiburg, Faculty of Medicine, University of Freiburg, Freiburg, Germany; 2 Department of Cardiology and Angiology I, Heart Center, University of Freiburg, Freiburg, Germany; 3 Institute of Experimental and Clinical Pharmacology and Toxicology, Faculty of Medicine, University of Freiburg, Freiburg, Germany; 4 Department of Medicine II, University Hospital Freiburg, Faculty of Medicine, University of Freiburg, Freiburg, Germany; 5 Department of Emergency Medicine, University Hospital of Freiburg, Faculty of Medicine, University of Freiburg, Freiburg, Germany; 6 Division of Experimental and Clinical Hemostasis, Hemotherapy, and Transfusion Medicine, Institute of Transplantation Diagnostics and Cell Therapy, Heinrich Heine University Medical Center, Düsseldorf, Germany; 7 Hemophilia Comprehensive Care Center, Institute of Transplantation Diagnostics and Cell Therapy, Heinrich Heine University Medical Center, Düsseldorf, Germany; 8 Department of Cardiology, Angiology, Haemostaseology and Medical Intensive Care, University Medical Center Mannheim, Medical Faculty Mannheim, Heidelberg University, Mannheim, Germany; 9 European Center for AngioScience and German Center for Cardiovascular Research partner site Heidelberg/Mannheim, Mannheim, Germany

**Keywords:** COVID-19, neutrophils, platelets, platelet aggregation, SARS-CoV-2

## Abstract

**Background:**

Conflicting results have been reported on platelet activity *ex vivo* and responsiveness *in vitro* among patients with COVID-19 with or without thromboembolic complications.

**Objectives:**

To assess platelet reactivity in patients with moderate disease at early stages of COVID-19.

**Methods:**

We performed a prospective, descriptive analysis of 100 consecutive patients presenting with suspected SARS-CoV-2 infection at University Medical Center Freiburg during the first or second wave of the pandemic. Following polymerase chain reaction testing and compliance with study inclusion criteria, 20 SARS-CoV-2–positive and 55 SARS-CoV-2–negative patients (serving as patient controls) were enrolled. In addition, 15 healthy subjects were included. Platelet reactivity was assessed using whole-blood impedance aggregometry and flow cytometry in response to various agonists.

**Results:**

Platelet aggregation was significantly impaired in the patients with COVID-19 compared with that in the patient controls or healthy subjects. The reduced platelet responsiveness in the patients with COVID-19 was associated with impaired activation of GPIIb/IIIa (α_IIb_β_3_). In contrast, low expression of P-selectin at baseline and intact secretion upon stimulation *in vitro* suggest that no preactivation *in vivo*, leading to “exhausted” platelets, had occurred. The proportion of circulating platelet-neutrophil complexes was significantly higher in the patients with COVID-19 (mean ± SD, 41% ± 13%) than in the patient controls (18% ± 7%; 95% CI, 11.1-34.1; *P* = .0002) or healthy subjects (17% ± 4%; 95% CI, 13.8-33.8; *P* < .0001). An analysis of neutrophil adhesion receptors revealed upregulation of CD11b (α-subunit of α_M_β_2_) and CD66b (CEACAM8) but not of CD162 (PSGL-1) in the patients with COVID-19.

**Conclusion:**

Despite reduced platelet responsiveness, platelet-neutrophil complexes are increased at early stages of moderate disease. Thus, this cellular interaction may occur during COVID-19 without preceding platelet activation.

## Essentials

• Both hyperreactive and hyporesponsive platelets have been described in patients with COVID-19.
• We studied platelet and neutrophil (re)activity during early SARS-CoV-2 infection.
• Neutrophils displayed hyperreactivity, whereas platelet reactivity was partially impaired.
• The number of platelet-neutrophil complexes was increased despite the absence of activated platelets.

## 1 Introduction

COVID-19 is a systemic but predominantly respiratory disease caused by SARS-CoV-2. The first case report from Wuhan indicated an association between pulmonary embolism and SARS-CoV-2 infection [1]. Since then, numerous trials have reported a high rate of venous and arterial thromboembolic events in patients with COVID-19, especially severe courses [[2], [3], [4], [5], [6]]. The underlying mechanisms leading to a prothrombotic state and severe COVID-19 are incompletely understood. It has been discussed that COVID-19–associated coagulopathy differs from other forms of infection-induced hemostatic changes resulting from the release of proinflammatory cytokines and a dysfunctional endothelium [[7], [8], [9]].

In general, inflammation and activation of coagulation represent concurrent responses of the host organism to contain and defend against invading pathogens. These multifaceted processes are referred to as “immunothrombosis” or “thromboinflammation” [10,11]. Neutrophils are essential components of innate immunity and are in the frontline of defense against infection, and formation of neutrophil extracellular traps (NETs) has been described to contribute to COVID-19 pathogenesis [12,13]. Moreover, platelets, being part of the innate immune system, can play an important role in viral infections [14,15]. Platelets are activated by NET formation (NETosis) [16] and express a variety of receptors, such as toll-like receptors, that detect viral pathogen-associated molecular patterns [17,18]. Stimulation of these receptors can also result in platelet activation, granular secretion, and aggregation.

Several studies investigating platelet reactivity in patients with COVID-19, specifically those with severe disease courses and thromboembolic events, have reported conflicting results. Some studies have suggested abnormally increased platelet function [[19], [20], [21], [22]], whereas others have described impaired platelet function during SARS-CoV-2 infection [[23], [24], [25], [26], [27]].

We here report that activation of neutrophils and formation of platelet-neutrophil complexes (PNCs) is a feature of the early phase in SARS-CoV-2 infection, whereas platelet reactivity can be reduced at this stage of the disease.

## 2 Methods

### 2.1 Study population

The data reported are part of an investigator-initiated, single-center prospective registry to study biomarkers associated with COVID-19 (Deutsches Register Klinische Studien, DRKS00021206); the study was conducted at University Medical Center Freiburg.

The protocol of the present study conforms to the ethical guidelines of the Helsinki Declaration and was approved by the Institutional Ethical Committee of Freiburg University (EK 153/20).

Patients admitted to the Department of Emergency Medicine of University Medical Center Freiburg because of suspected or proven infection with SARS-CoV-2 were eligible for this study. The decision to perform a polymerase chain reaction (PCR) test for SARS-CoV-2 in deep oropharyngeal or nasopharyngeal swabs was made independently of study inclusion by treating physicians. Patients who were or had been tested positive for SARS-CoV-2 within the last 2 weeks were allocated to the positive group; negatively tested patients served as the patient control group. Healthy volunteers (recruited from medical staff and students) served as an additional control group.

Upon informed, written consent by patients and volunteers, hirudinized whole blood (S-Monovette Hirudin 1.6 mL; Sarstedt) was collected on the day of inclusion to perform whole-blood impedance aggregometry (WBIA; Multiplate, Roche Diagnostics, Roche) and flow cytometric analyses. Laboratory parameters were determined by the Freiburg central laboratory (Table). The von Willebrand factor (VWF) antigen was determined immunoturbidimetrically (VWF Ag test kit; Siemens Healthcare Diagnostics Products). For assessment of VWF activity, binding of VWF to GPIb in the absence of ristocetin was measured turbidimetrically (INNOVANCE VWF Ac; Siemens Healthcare Diagnostics Products). Patient characteristics, including medical history, clinical symptoms, or previous medication, were analyzed. The severity of illness was assessed in all patients using the Sequential Organ Failure Assessment score [28].

**Table:** Patient characteristics in comparison with healthy volunteers and laboratory parameters.

Parameter	SARS-CoV-2–positive patients (n = 20)	SARS-CoV-2–negative patients (n = 55)	Healthy volunteers (n = 15)	95% CI *P* value Positive vs negative	95% CI *P* value Positive vs healthy	95% CI *P* value Negative vs healthy
**At admission**						
Male	11 (55.0%)	32 (58.2%)	6 (40.0%)	.81^a^	.38^a^	.21^a^
Age (y)	62 (46-81)	66 (49-77)	23 (21-26)	>.99^b^	<.0001^b^	<.0001^b^
Erythrocytes (× 10^6^/μL)	4.4 ± 0.6	4.3 ± 0.6	4.8 ± 0.5	−0.2 to 0.5>.99^c^	−0.9 to 0.2.28^c^	−0.9 to −0.1.02^c^
Hemoglobin (g/dL)	13.4 ± 1.6	12.7 ± 1.9	14.2 ± 1.5	−0.4 to 1.8.40^c^	−2.2 to 0.7.67^c^	−2.7 to −0.2.02^c^
Hematocrit (%)	38.1 ± 4.0	36.8 ± 5.1	40.3 ± 4.1	−1.7 to 4.3.91^c^	−6.1 to 1.8.56^c^	−6.8 to −0.1.04^c^
Platelets (× 10^3^/μL)	179.9 ± 51.3	235.7 ± 73.6	232.4 ± 36.8	−92.5 to −19.2.002^d^	−89.1 to −16.0.004^d^	−29.9 to 36.5.97^d^
Leukocytes (× 10^3^/μL)	4.7 ± 1.8	9.1 ± 4.5	6.1 ± 1.4	−6.1 to −2.6<.0001^d^	−2.7 to −0.03.04^d^	1.3-4.7.0002^d^
Fibrinogen Clauss (mg/dL)^f^	416.4 ± 131.6	454.6 ± 186.1	231.1 ± 54.2	−138.9 to 62.6.63^d^	102.5-268.3<.0001^d^	146.5-300.5<.0001^d^
VWF antigen (%)^f^	288.2 ± 126.9	250.4 ± 141.3	97.9 ± 28.3	−45.8 to 121.4.52^d^	116.6-264.0<.0001^d^	103.1-201.9<.0001^d^
VWF activity (%)^f^	247.0 ± 91.0	247.3 ± 135.8	98.2 ± 27.2	−66.7 to 66.1>.99^d^	94.9-202.7<.0001^d^	101.7-196.5<.0001^d^
Dyspnea	9 (45.0%)	31 (56.4%)		.38^a^		
Fever	14 (70.0%)	27 (49.1%)		.11^a^		
Cough	13 (65.0%)	19 (34.5%)		.02^a^		
Oxygen requirement	6 (30.0%)	19 (34.5%)		.71^a^		
SOFA score^f^	1 (0-3)	0 (0-2)		.16^e^		
D-dimers (mg/dL)^f^	0.7 (0.4-1.3)	0.7 (0.3-2.0)	<0.5^g^	.75^e^		
**Upon30-dfollow-up**						
Non-ICU inpatients	16 (80.0%)	35 (63.6%)		.18^a^		
ICU inpatients	1 (5.0%)	4 (7.3%)		.73^a^		
Hospitalization (d)	9 (8-22)	10 (6-15)		.60^e^		
Thrombotic events	2 (10.0%)	2 (3.6%)		.28^a^		
Bleeding	0 (0.0%)	1 (1.8%)		.54^a^		
Death^f^	0 (0.0%)	4 (7.7%)		.23^a^		
**Cause ofhospitalization**						
Infectious disease^h^		30 (54.5%)				
Heart disease		6 (10.9%)				
Cancer		5 (9.1%)				
Other disorders		14 (25.5%)				

Values are provided as mean ± SD in case of normal distribution or as median with interquartile range in case of nonnormal distribution.

ICU, intensive care unit; SOFA, Sequential Organ Failure Assessment.

a Based on the chi-square test.

b Based on the Kruskal-Wallis test and Dunn multiple comparison test.

c Based on 1-way analysis of variance with the Bonferroni multiple comparisons test.

d Based on Welch analysis of variance with the Games-Howell multiple comparisons test.

e Based on the Mann–Whitney test.

f Value not available from every subject: SOFA score: SARS-CoV-2 positive n = 17, SARS-CoV-2 negative n = 53; fibrinogen: SARS-CoV-2 positive n = 19, SARS-CoV-2 negative n = 42; von Willebrand factor antigen or von Willebrand factor activity: SARS-CoV-2 negative n = 54; D-dimers: SARS-CoV-2 positive n = 13, SARS-CoV-2 negative n = 41; death: SARS-CoV-2 positive n = 18, SARS-CoV-2 negative n = 52.

g Reference range of Freiburg central laboratory.

h Other than SARS-CoV-2; non-ICU inpatients: patients receiving non-ICU ward care; patients with infectious disease: respiratory infection, pneumonia, pleurisy, influenza, tonsillitis, peritonsillitis, esophagitis, cholangitis, neutrocytopenic fever, urinary tract infection, infected lymphocele, erysipelas, viral meningitis, cholangiosepsis, urosepsis, septic shock; patients with heart disease: cardiac decompensation, hydropic decompensation, heart failure with preserved ejection fraction, terminal heart failure; patients with cancer: lung cancer, adrenocortical carcinoma, cancer of unknown primary origin; patients with other disorders: gait disorder, chest pain, hemolytic anemia, headache, migraine attack, pulmonary hypertension, chronic obstructive lung disease, dyspnea, pulmonary sarcoidosis.

Patients were enrolled from April 7 through May 22 and from October 19 through December 18, 2020, ie, during the first and second wave of the SARS-CoV-2 pandemic in Germany.

**Figure 1:**
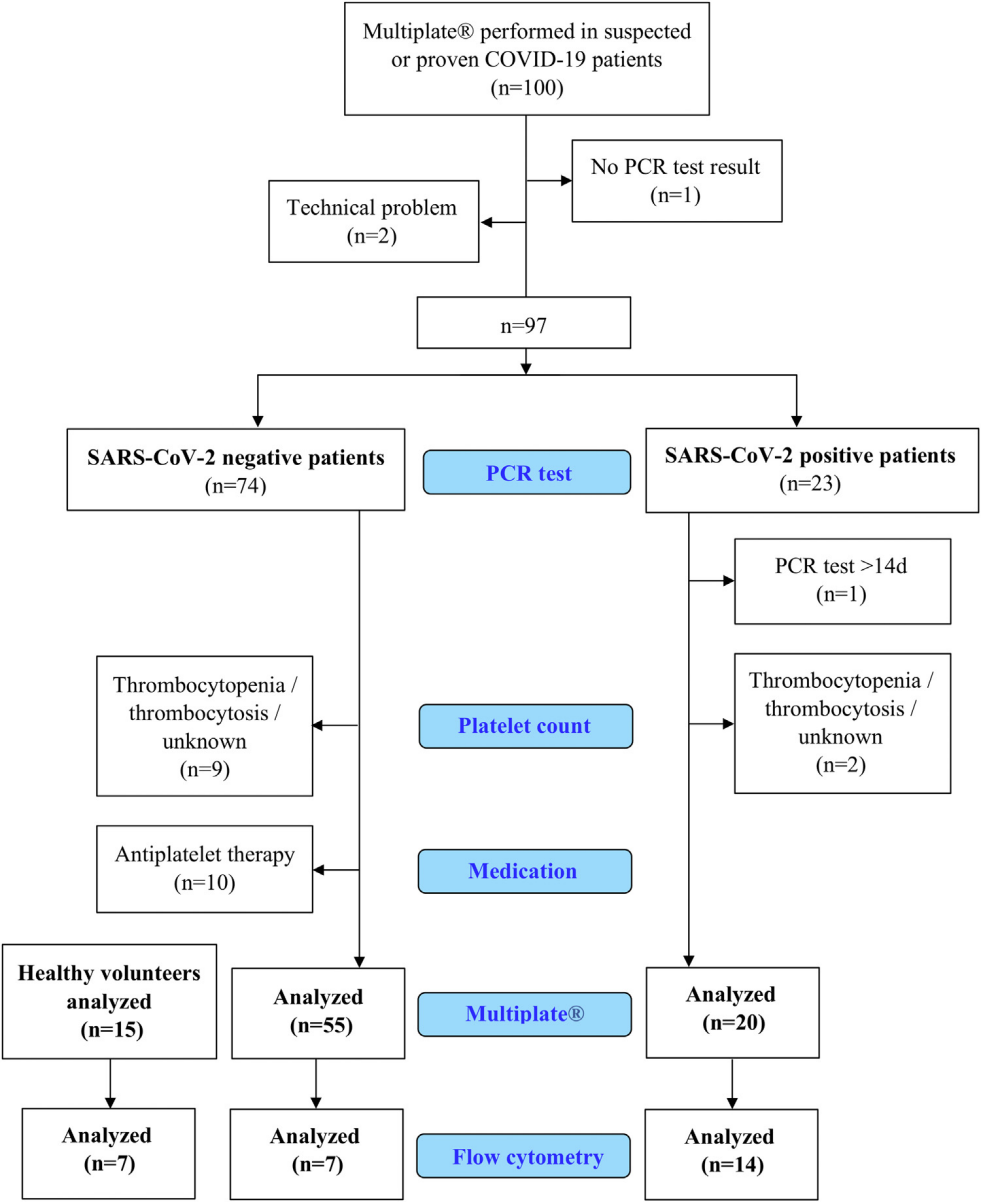
Allocation scheme of the study population. We performed whole-blood impedance aggregometry (Multiplate) on a total of 100 patients who had been admitted to the Emergency Department of University Medical Center Freiburg and had a confirmed SARS-CoV-2 infection or were suspected to have COVID-19 based on their presentation with typical symptoms (fever, cough, and/or dyspnea). We excluded 1 patient because of a missing polymerase chain reaction (PCR) test result and 2 patients because of technical problems during Multiplate analysis. Based on PCR testing, 23 and 74 patients were allocated to the SARS-CoV-2–positive and SARS-CoV-2–negative group, respectively. We had to exclude 1 SARS-CoV-2–positive patient with a PCR test result more than 2 weeks ago and a negative test result at admission, 2 SARS-CoV-2–positive patients, and 9 SARS-CoV-2–negative patients with platelet counts <100 × 10^3^/μL or >450 × 10^3^/μL or when the platelet count was not available. Ten other SARS-CoV-2–negative patients had to be excluded because of aspirin medication. Eventually, 20 SARS-CoV-2–positive and 55 SARS-CoV-2–negative patients were included in the aggregometry analysis, and 14 SARS-CoV-2–positive and 7 SARS-CoV-2–negative patients were included in the flow cytometry study. For comparison (control), 15 healthy subjects were studied using aggregometry and 7 of them using flow cytometry

WBIA (Multiplate) was performed in all 100 patients with suspected or proven COVID-19 (Figure 1). The exclusion procedure is described in Figure 1. Eventually, data of 20 SARS-CoV-2–positive and 55 SARS-CoV-2–negative patients were used for the aggregometry analysis. Only during the second wave of the pandemic, flow cytometry studies were parallelly conducted in all 14 SARS-CoV-2–positive and 7 SARS-CoV-2–negative patients. The findings obtained in the patients were compared with those of the healthy volunteers (15 aggregometry and 7 flow cytometric analyses).

The SARS-CoV-2–positive patients were predominantly in the early phase of the infection (7 tested positive for the first time at inclusion, 11 tested positive between day 1 and 9 before inclusion, and 2 tested positive between day 10 and 30 before inclusion) (Supplementary Table).

### 2.2 WBIA

We used WBIA (Multiplate Analyzer, Roche Diagnostics) according to the manufacturer’s instructions, respecting the limitations described in the package insert. Platelet aggregation was tested in response to thrombin receptor-activating peptide 6 (TRAP-6, 32 μM; TRAPtest, Roche Diagnostics), adenosine diphosphate (ADP, 6.5 μM; ADPtest, Roche Diagnostics), or arachidonic acid (AA, 0.5 mM; ASPItest, Roche Diagnostics). The results were expressed as area under the curve (AUC) in arbitrary units (U).

### 2.3 Flow cytometry

Flow cytometry was used to determine surface activation markers of platelets (P-selectin, activated GPIIb/IIIa) and of neutrophils (CD11b, CD162, CD66b), and PNCs. Hirudinized whole blood was diluted in a ratio of 1:6 with Dulbecco phosphate-buffered saline (DPBS) with Ca^2+^ and Mg^2+^ and supplemented with 525 antithrombin units/mL of recombinant hirudin (Hyphen BioMed) to prevent dilution of the anticoagulant. The samples were incubated with TRAP-6 (20 μM; Abcam), ADP (20 μM; MoeLab), phorbol 12-myristate 13-acetate (PMA 100 nM; Sigma-Aldrich), or DPBS as control (15 minutes, room temperature [RT]). Subsequently, the samples were separately incubated (15 minutes, RT, in the dark) with 3 specific antibody mixes (or respective isotype controls) to stain for platelet activation markers (HIP1 clone mouse antihuman CD42b-PE, AK4 clone mouse antihuman CD62P-PE Cy7, and PAC-1 clone mouse antihuman CD41/CD61-AF647), neutrophil activation markers (HI30 clone mouse antihuman CD45-APC, M1/70 clone mouse antihuman CD11b-PE, KPL-1 clone mouse antihuman CD162-PE Cy7, and G10F5 clone mouse antihuman CD66b-PerCP/Cy5.5), or PNCs (HI30 clone mouse antihuman CD45-Pacific Blue and HIP1 clone mouse antihuman CD42b-PE). All the antibodies were purchased from BioLegend. Afterward, lysis of red blood cells and fixation were simultaneously performed by incubation (30 minutes, RT) with prewarmed (37 °C) phosflow lyse and fix buffer (BD Biosciences). Finally, the samples were centrifuged (700 *g*, 5 minutes, RT), resuspended in DPBS, and analyzed using flow cytometry (FACSCanto II; BD). The percentage of positive cells was determined by appropriate gating using isotype controls. Antibody binding was also quantified based on geometric mean fluorescent intensity (GMFI).

### 2.4 Statistical analysis

Statistical analyses were performed using GraphPad Prism (version 8; GraphPad Software). The normal distribution and homogeneity of variances were tested using the Shapiro-Wilk test and Bartlett test, respectively. Data are presented as mean ± SD in case of normal distribution or as median with interquartile range in case of nonnormal distribution. For comparison of 3 groups and normal distribution with homogeneity of variance, we used parametric 1-way analysis of variance with the Bonferroni multiple comparison test. When homogeneity of variances was absent, we applied the Welch analysis of variance with the Games-Howell multiple comparison test. When data were not normally distributed, the nonparametric Mann–Whitney test for the comparison of 2 groups or nonparametric Kruskal-Wallis test with the Dunn multiple comparison test for comparison of 3 groups was performed. For categorical variables, we used the chi-square test. A 2-tailed *P* value of <.05 was considered statistically significant. The 95% CI is provided for differences in means among the groups.

## 3 Results

### 3.1 Characteristics of the study population

The SARS-CoV-2–positive and SARS-CoV-2–negative patients displayed a similar sex ratio or demographic and clinical profile (Table). Likewise, the fibrinogen (Clauss) and VWF plasma levels did not differ between the SARS-CoV-2–positive and SARS-CoV-2–negative patients but were significantly higher in both the patient groups than in the healthy controls (*P* < .0001). In contrast, the mean platelet count was lower in the SARS-CoV-2–positive patients than in the SARS-CoV-2–negative patients (*P* = .002) or healthy subjects (*P* = .004). The leukocyte count in the SARS-CoV-2–infected individuals was decreased compared with that in both the control groups (*P* < .0001 and *P* = .04, respectively).

The majority of the patients had a mild-to-moderate disease course. Most of them were hospitalized; however, only a few required intensive care. Both the patient groups presented with similar symptoms (dyspnea, fever, and/or cough), whereby the percentage of patients with cough was significantly higher in the SARS-CoV-2–positive group than in the SARS-CoV-2–negative group (*P* = .02). Approximately one-third of each patient group required oxygen, and the Sequential Organ Failure Assessment scores were similarly low. The patients were followed up on day 30. Venous thromboembolism was rare, with 2 venous thromboembolism events in each patient group. This is in line with low D-dimer levels in both the patient groups (median, <1 mg/dL; reference range, <0.5 mg/dL). Bleeding was observed in 1 SARS-CoV-2–negative patient. No lethal outcome occurred in the SARS-CoV-2–positive group, whereas 4 SARS-CoV-2–negative patients died.

Most of the SARS-CoV-2–negative patients had infectious diseases (54.5%), followed by various other disorders (25.5%), heart disease (10.9%), and cancer (9.1%).

### 3.2 Platelet aggregation is reduced in SARS-CoV-2–positive patients

**Figure 2:**
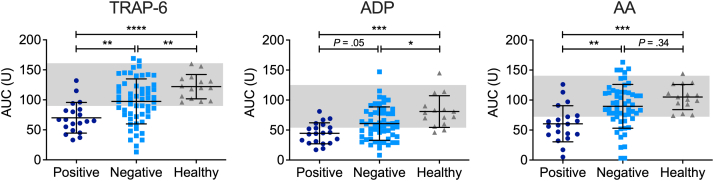
Agonist-induced platelet aggregation is reduced in SARS-CoV-2–positive patients. Platelet aggregation was analyzed using whole blood impedance aggregometry (Multiplate) using hirudinized whole blood (1:2 diluted) from SARS-CoV-2–positive patients (dark blue circles, n = 20), SARS-CoV-2–negative patients (light blue squares, n = 55), and healthy volunteers (grey triangles, n = 15). Platelet aggregation was induced by stimulation with thrombin receptor-activating peptide 6 (32 μM), adenosine diphosphate (6.5 μM), or arachidonic acid (0.5 mM) and expressed as area under the curve in arbitrary units. The shaded area represents the reference range, as provided by the manufacturer. Data presented are individual values and mean ± SD of each group. Data were analyzed using 1-way analysis of variance with the Bonferroni multiple comparisons test or Welch analysis of variance with the Games-Howell multiple comparisons test. ∗*P* < .05; ∗∗*P* < .01; ∗∗∗*P* < .001; ∗∗∗∗*P* < .0001. AA, arachidonic acid; ADP, adenosine diphosphate; AUC, area under the curve; TRAP-6, thrombin receptor-activating peptide 6

First, we were interested to determine whether early infection with SARS-CoV-2 is associated with changes in platelet aggregation responses upon stimulation *in vitro*. The AUC values of the healthy subjects were within the reference range, represented by the shaded area in Figure 2 (TRAP-6, 94-156 U; ADP, 53-122 U; AA, 75-136 U). Notably, most SARS-CoV-2–positive patients displayed AUC values below the reference range, with a significant decrease compared with the healthy controls in response to TRAP-6 (CI, −70.8 to −32.9; *P* < .0001), ADP (CI, −57.7 to −14.9; *P* = .0002), or AA (CI, −72.2 to −16.9; *P* = .0005).

The aggregation responses of the SARS-CoV-2–positive group were not only lower than those of the healthy controls but also decreased compared with those of the SARS-CoV-2–negative patients after incubation with TRAP-6 (CI, −45.7 to −8.9; *P* = .002), ADP (CI, −32.6 to 0.2; *P* = .05), or AA (CI, −50.3 to −8.0; *P* = .003).

Of the 55 SARS-CoV-2–negative patients, 25 (45.5%), 23 (41.8%), and 15 (27.3%) had AUC values below the reference range in response to TRAP-6, ADP, and AA, respectively. The aggregation responses were lower than those of the healthy controls upon stimulation with TRAP-6 (CI, −42.2 to −6.9; *P* = .004), ADP (CI, −38.3 to −1.8; *P* = .03), or AA (CI, −39.0 to 8.2; *P* = .34).

### 3.3 Platelet α-granule secretion is not affected in SARS-CoV-2–positive patients

**Figure 3:**
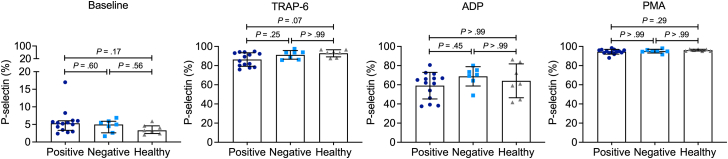
Platelet P-selectin surface expression is not affected in SARS-CoV-2–positive patients. P-selectin on the platelets surface was assessed in hirudinized whole blood (1:6 diluted) from SARS-CoV-2–positive patients (dark blue circles, n = 14), SARS-CoV-2–negative patients (light blue squares, n = 7), and healthy volunteers (grey triangles, n = 7) after incubation with phosphate-buffered saline (baseline), thrombin receptor-activating peptide 6 (20 μM), adenosine diphosphate (20 μM), or phorbol 12-myristate 13-acetate (100 nM) for 15 minutes using flow cytometry. P-selectin expression was determined as the percentage of positive platelets. Data presented are individual values and mean ± SD of each group. Data were analyzed using 1-way analysis of variance with the Bonferroni multiple comparisons test or Welch analysis of variance with the Games-Howell multiple comparisons test. ADP, adenosine diphosphate; PMA, phorbol 12-myristate 13-acetate; TRAP-6, thrombin receptor-activating peptide 6

Next, we examined whether reduced platelet aggregation responsiveness upon stimulation *in vitro* results from circulating “exhausted” platelets because of increased activation *in vivo*. Such a phenomenon has been described in a variety of clinical conditions [[29], [30], [31]] and also in patients with COVID-19 [32]. We measured the surface expression of P-selectin, an established marker of platelet activation and an indicator of α-granule secretion. In fact, we did not detect platelet preactivation because the percentage of circulating P-selectin–positive platelets was low at baseline in the SARS-CoV-2–positive patients (5.7% ± 3.6%) and similar compared with that in the SARS-CoV-2–negative patients (4.6% ± 1.9%) and healthy subjects (3.7% ± 1.3%) (Figure 3). Moreover, platelet α-granule secretion was intact upon thrombin receptor stimulation (a mean of >85% positive in all the groups) or ADP receptor activation (a mean of >55% positive in all the groups). Complete surface expression of P-selectin was achieved following receptor-independent stimulation with the direct protein kinase C activator PMA (a mean of >90% positive in all the groups).

### 3.4 Activation of platelet GPIIb/IIIa is partially impaired in SARS-CoV-2–positive patients

**Figure 4:**
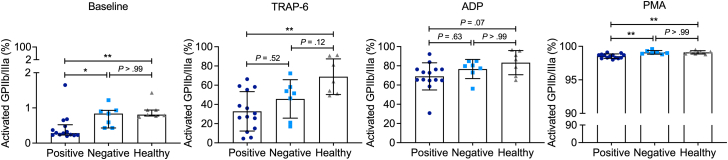
Platelet GPIIb/IIIa activation is partially impaired in SARS-CoV-2–positive patients. Activated GPIIb/IIIa on the platelets surface was assessed in hirudinized whole blood (1:6 diluted) from SARS-CoV-2–positive patients (dark blue circles, n = 14), SARS-CoV-2–negative patients (light blue squares, n = 7), and healthy volunteers (grey triangles, n = 7) after incubation with phosphate-buffered saline (baseline), thrombin receptor-activating peptide 6 (20 μM), adenosine diphosphate (20 μM), or phorbol 12-myristate 13-acetate (100 nM) for 15 minutes using flow cytometry in combination with PAC-1, an activation-dependent monoclonal antibody. Expression of activated GPIIb/IIIa was determined as the percentage of PAC-1–positive platelets. Data presented are individual values and mean ± SD (thrombin receptor-activating peptide 6, adenosine diphosphate, phorbol 12-myristate 13-acetate) or median with interquartile range (baseline) of each group. Data were analyzed using 1-way analysis of variance with the Bonferroni multiple comparisons test or Kruskal-Wallis test. ∗*P* < .05; ∗∗*P* < .01. ADP, adenosine diphosphate; PMA, phorbol 12-myristate 13-acetate; TRAP-6, thrombin receptor-activating peptide 6

We then assessed activated GPIIb/IIIa (integrin α_IIb_β_3_) as another platelet activation parameter. In accordance with minor surface expression of P-selectin at the baseline (Figure 3), the percentage of platelets displaying activated GPIIb/IIIa was negligible at the baseline (a median of <1% in all the groups) (Figure 4). Notably, upon stimulation with TRAP-6, the percentage of platelets displaying activated GPIIb/IIIa in the SARS-CoV-2–positive patients (32.8% ± 20.5%) was lower than that in the SARS-CoV-2–negative patients (45.7% ± 20.0%; CI, −36.6 to 10.8; *P* = .52) and significantly reduced in comparison with that of the healthy individuals (68.9% ± 18.6%; CI, −59.7 to −12.4; *P* = .002). In response to ADP, there was a trend toward a lower percentage of activated GPIIb/IIIa in the SARS-CoV-2–positive patients (69.0% ± 14.1%) than in the healthy subjects (83.3% ± 12.6%; CI, −29.6 to 1.0; *P* = .07). More than 95% of platelets expressed activated GPIIb/IIIa upon stimulation with PMA in each of the 3 groups.

### 3.5 PNCs are elevated in SARS-CoV-2–positive patients

**Figure 5:**
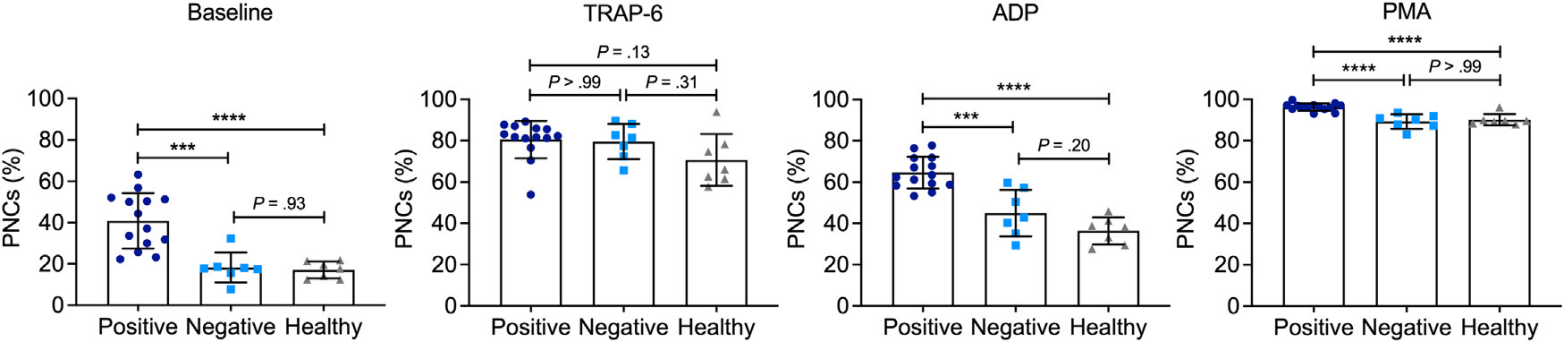
Platelet-neutrophil complexes are increased in SARS-CoV-2–positive patients. Platelet-neutrophil complexes were assessed in hirudinized whole blood (1:6 diluted) from SARS-CoV-2–positive patients (dark blue circles, n = 14), SARS-CoV-2–negative patients (light blue squares, n = 7), and healthy volunteers (grey triangles, n = 7) after incubation with phosphate-buffered saline (baseline), thrombin receptor activating peptide 6 (20 μM), adenosine diphosphate (20 μM), or phorbol 12-myristate 13-acetate (100 nM) for 15 minutes using flow cytometry. Platelet-neutrophil complexes were determined as the percentage of CD42b-positive neutrophils. Data presented are individual values and mean ± SD of each group. Data were analyzed using 1-way analysis of variance with the Bonferroni multiple comparisons test or Welch analysis of variance with the Games-Howell multiple comparisons test. ∗∗∗*P* < .001; ∗∗∗∗*P* < .0001. ADP, adenosine diphosphate; PMA, phorbol 12-myristate 13-acetate; PNCs, platelet-neutrophil complexes; TRAP-6, thrombin receptor-activating peptide 6

Activated platelets can bind to neutrophils, resulting in PNCs. Thus, circulating PNCs are another indicator of activated platelets *in vivo* [33]. Indeed, we detected a significantly higher percentage of PNCs at the baseline in the SARS-CoV-2–positive patients (40.9% ± 13.4%) versus that in the SARS-CoV-2–negative patients (18.2% ± 7.3%; CI, 11.1-34.1; *P* = .0002) and versus that in the healthy subjects (17.1% ± 4.1%; CI, 13.8-33.8; *P* < .0001) (Figure 5). Stimulation with TRAP-6 resulted in a similar increase in PNCs across all the groups, as demonstrated by the percentage of neutrophils with bound platelets (a mean of >70% in all the groups). In contrast, in response to ADP, the proportion of PNCs formed in the SARS-CoV-2–positive patients (64.6% ± 7.7%) was higher than that in the SARS-CoV-2–negative patients (45.0% ± 11.3%; CI, 9.5-29.6; *P* = .0001) or healthy volunteers (36.4% ± 6.6%; CI, 18.2-38.3; *P* < .0001). PMA most effectively induced PNCs in all the groups, with a higher increase in the percentage in the SARS-CoV-2–positive group (96.2% ± 1.8%) than the SARS-CoV-2–negative group (89.2% ± 3.5%; CI, 4.0-10.0; *P* < .0001) or healthy individuals (90.1% ± 2.8%; CI, 3.1-9.1; *P* < .0001).

### 3.6 Neutrophils are activated and hyperreactive in SARS-CoV-2–positive patients

**Figure 6:**
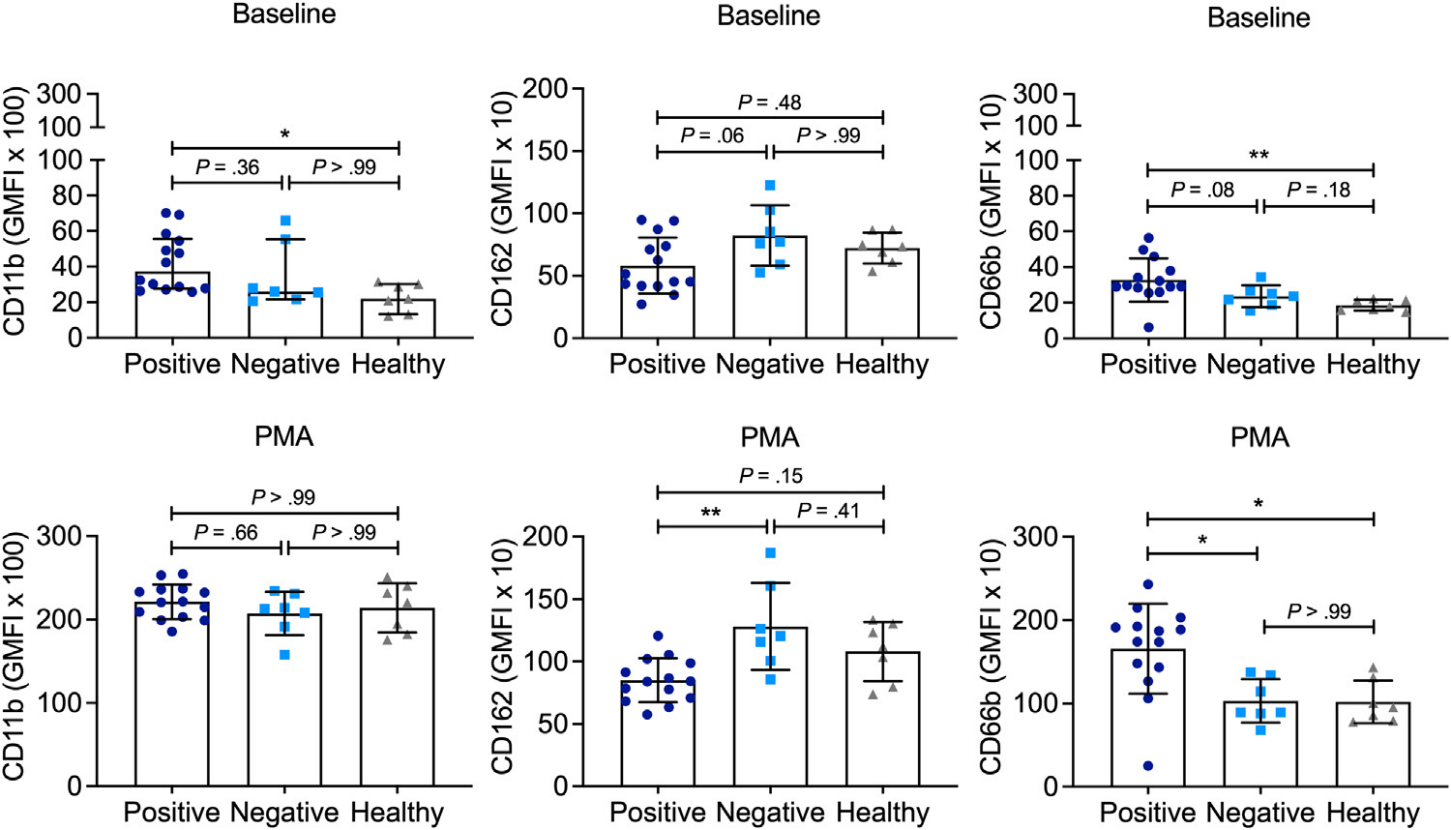
Neutrophil activation is elevated in SARS-CoV-2–positive patients. Surface expression of neutrophil activation markers CD11b (left panels), CD162 (middle panels), and CD66b (right panels) was assessed in hirudinized whole blood (1:6 diluted) from SARS-CoV-2–positive patients (dark blue circles, n = 14), SARS-CoV-2–negative patients (light blue squares, n = 7), and healthy volunteers (grey triangles, n = 7) after incubation with phosphate-buffered saline (baseline; upper panels) or phorbol 12-myristate 13-acetate (PMA, 100 nM; lower panels) for 15 minutes using flow cytometry and quantified based on geometric mean fluorescent intensity. Data presented are individual values and mean ± SD (CD11b PMA, CD162 baseline and PMA, and CD66b baseline and PMA) or median with interquartile range (CD11b baseline) of each group. Data were analyzed using 1-way analysis of variance with the Bonferroni multiple comparisons test or Welch analysis of variance with the Games-Howell multiple comparisons test or Kruskal-Wallis test. ∗*P* < .05; ∗∗*P* < .01.

The increased number of circulating PNCs in patients with COVID-19 (Figure 5) was surprising because P-selectin (Figure 3) and activated GPIIb/IIIa (Figure 4) were not increasingly expressed on the platelet surface at the baseline. Both the glycoproteins can mediate binding to neutrophils either directly via PSGL-1 (CD162) or by ligation of plasma fibrinogen to form bridges with Mac-1 (CD11b/CD18; integrin α_M_β_2_) [34]. Therefore, we examined whether upregulation of neutrophil adhesion molecules in response to SARS-CoV-2 infection could be a possible explanation. Indeed, we found increased surface expression of CD11b in the SARS-CoV-2–positive patients (Figure 6). The GMFI of CD11b tended to be higher in the SARS-CoV-2–positive (3734.5 [2769.5-5553.0] arbitrary units [AU]) than in the SARS-CoV-2–negative patients (2615.0 [2169.0-5535.0] AU; *P* = .36) and was significantly increased in the SARS-CoV-2–positive patients in comparison with that in the healthy volunteers (2196.0 [1333.0-3019.0] AU; *P* = .04) at the baseline. However, the surface expression of CD11b in response to PMA was not different between the groups. In contrast to CD11b, the surface expression of CD162 tended to be lower in the patients with COVID-19 (581.6 ± 224.2 AU) than in the patient controls (821.1 ± 242.3 AU; CI, −488.5 to 9.4; *P* = .06) or healthy subjects (722.3 ± 124.3 AU; CI, −389.7 to 108.2; *P* = .48). This observation suggests that CD162 shedding in the patients with COVID-19 had occurred *in vivo*, which is a known response to leukocyte activation [35].

Decreased surface expression of CD162 was also detected upon activation *in vitro* with PMA (GMFI, SARS-CoV-2 positive: 849.5 ± 175.5 AU vs SARS-CoV-2 negative: 1280.1 ± 348.2 AU; CI, −718.3 to −143.0; *P* = .002 vs healthy: 1080.6 ± 237.3 AU; CI, −518.7 to 56.6; *P* = .15). Remarkably, the surface expression of CD66b (CEACAM8), which has not yet been described to have a major role in PNC formation, was upregulated in the SARS-CoV-2–positive patients (GMFI, SARS-CoV-2 positive: 327.9 ± 121.3 AU vs SARS-CoV-2 negative: 236.6 ± 60.4 AU; CI, −9.4 to 192.1; *P* = .08 vs healthy: 186.3 ± 30.3 AU; CI, 52.8-230.4; *P* = .002). Moreover, stimulation with PMA induced stronger CD66b expression on the surface of neutrophils from the SARS-CoV-2–positive patients (1655.5 ±540.4 AU), with a significant increase, compared with that in both the SARS-CoV-2–negative patients (1029.4 ± 261.4 AU; CI, 116.4–1136.0; *P* = .01) and healthy controls (1020.0 ± 257.1 AU; CI, 125.8-1145.0; *P* = .01). Taken together, these data indicate that neutrophil reactivity is increased during early SARS-CoV-2 infection.

## 4 Discussion

The present study demonstrates that the proportion of circulating PNCs *ex vivo* is increased in patients with mild-to-moderate disease at early stages of COVID-19 (Figure 5). In contrast, healthy volunteers and patients with similar clinical features of other pathologies do not display signs of platelet-neutrophil interaction. Thus, the increased number of PNCs appears to be a characteristic feature of SARS-CoV-2 infection. This finding is in accordance with the role of neutrophils in innate immunity as frontline defenders against invading pathogens. Moreover, our observation is consistent with results from other investigators who reported elevated numbers of PNCs in intensive care unit (ICU) and non-ICU patients with COVID-19 in comparison with those in healthy subjects [21,[36], [37], [38], [39], [40], [41]]. Canzano et al. [36] also found a higher number of PNCs in patients with COVID-19 upon comparison with that in patients with coronary artery disease.

The “classical” mechanism by which PNCs are generated is thought to be exclusively initiated by activated platelets [42], and platelet surface expression of P-selectin is a major driver of PNC formation [43]. In line with this, most of the aforementioned studies reported increased numbers of circulating PNCs in parallel with upregulated platelet surface expression of P-selectin. However, the P-selectin expression at the baseline was negligibly low in the SARS-CoV-2–positive patients in the present study (Figure 3), and this is an unlikely explanation for the observed increased number of PNCs.

We, therefore, investigated whether the upregulation of neutrophil adhesion molecules, such as integrin receptor Mac-1 (α_M_β_2_; CD11b/CD18), PSGL-1 (CD162), or CAECAM8 (CD66b), could have mediated PNC formation in response to SARS-CoV-2 infection at early stages of mild-to-moderate COVID-19. Indeed, as depicted in Figure 6, the surface expression of CD11b and CD66b (but not of CD162) was significantly increased in the SARS-CoV-2–positive patients at the baseline in comparison with that in the healthy volunteers. In accordance with our data, recent reports have described upregulation of CD11b [38] or CD66b [39] on neutrophils, together with a concomitant increase in the number of PNCs in patients with mild-to-moderate COVID-19. CD11b is part of the integrin receptor Mac-1 (α_M_β_2_; CD11b/CD18). Because Mac-1 is known to be a direct binding partner of platelet GPIb [44], this interaction could explain PNC formation during early SARS-CoV-2 infection, even in the absence of platelet activation. Whether CD66b contributes to platelet-neutrophil interaction and which putative ligand on the platelet surface might be involved are currently unknown. Moreover, in addition to neutrophil activation at the baseline, we observed neutrophil hyperreactivity in the SARS-CoV-2–positive patients, as demonstrated by increased surface expression of CD66b and increased shedding of CD162 in response to PMA stimulation (Figure 6). However, because PMA is a general cellular activator, we cannot distinguish whether PMA-induced changes in neutrophil surface expression in the SARS-CoV-2–positive group were due to hyperreactivity of neutrophils only or also mediated by activated platelets.

To assess PNC formation among patients at an early stage of SARS-CoV-2 infection in more detail, we studied the response to various stimuli *in vitro*. Incubation of anticoagulated whole blood with TRAP-6 further increased the number of PNCs (Figure 5). However, this stimulation *in vitro* had a similar effect on the number of PNCs in the patients with and without COVID-19 and healthy volunteers; so, the differences recorded at the baseline disappeared. This can be explained by TRAP-6–induced P-selectin expression on platelets, which was similarly increased across all the study groups. Thus, it is likely that PNC formation is mediated by P-selectin under this experimental condition.

In contrast, incubation of whole blood with PMA caused a significant increase in the number of PNCs among the SARS-CoV-2–positive patients in comparison with that in the patient controls and healthy volunteers (Figure 5). PMA is a potent stimulus of both neutrophils and platelets. The stimulation of whole blood with PMA resulted in maximal platelet surface expression of P-selectin across all the groups (Figure 3), whereas the neutrophil surface expression of CD66b in the patients with SARS-CoV-2 infection was significantly upregulated in comparison with that in the patient controls and healthy subjects (Figure 6). Thus, PMA-induced PNC formation *in vitro* is likely a consequence of both platelet surface expression of P-selectin and upregulation of neutrophil surface adhesins.

Of note, the stimulation of whole blood with ADP resulted in a significantly increased number of PNCs in the patients with COVID-19 in comparison with that in the patient controls and healthy volunteers (Figure 5). This observation requires distinct consideration. One could assume that this ADP-induced effect might have resulted from concurrent platelet activation, leading to a higher proportion of PNCs. However, ADP is a weak platelet agonist and induced P-selectin expression on platelets, without significant differences among all the groups (Figure 3). Moreover, such an explanation suggesting a platelet-induced effect would not be consistent with the findings obtained upon exposure to TRAP-6. On the other hand, in comparison with TRAP-6, incubation with ADP resulted in a higher percentage of platelets with GPIIb/IIIa activation (Figure 4). Constantinescu-Bercu et al. [45] recently showed that activated GPIIb/IIIa binds to SLC44A2 on neutrophils, which could contribute to PNC formation. However, GPIIb/IIIa activation upon stimulation with ADP was not increased in the patients with COVID-19 compared with that in the controls (Figure 4). In addition, as discussed below, the low platelet aggregation responsiveness in patients with mild-to-moderate COVID-19 makes it rather unlikely that the effect of ADP on PNCs is mediated by platelet activation. A more conceivable explanation would be direct stimulation of neutrophils by ADP via purinergic receptors. Such a contention is supported by studies demonstrating the expression of a P2Y12 receptor on leukocytes [46], specifically on eosinophils [47].

Altogether, our findings suggest that PNCs at early stages of SARS-CoV-2 infection are initiated by activated neutrophils. Such a contention underlines the role of neutrophils as “frontline defenders” against invading pathogens. However, the “traditional” concept regarding PNC formation suggests that activated platelets interact with neutrophils, whereby platelet activation is considered an essential requirement in this cellular interplay. Along with our study results, we now propose that increased platelet activity is not a mandatory prerequisite for the formation of circulating PNCs during COVID-19. This conclusion is based on several observations reported here.

First, we observed a reduced platelet aggregation response in patients with mild-to-moderate COVID-19 (Figure 2). This is consistent with previous studies that performed WBIA to test platelet function. Bertolin et al. [23] demonstrated lower platelet reactivity using WBIA in hospitalized patients with nonsevere COVID-19. However, reduced aggregation responses were not limited to a nonsevere clinical course of COVID-19. Heinz et al. [24] also observed normal or reduced aggregation responses using WBIA in ICU patients with COVID-19. A longitudinal assessment by another group revealed that platelet aggregation remained reduced even after 14 days of admission to the ICU [25]. Of note, impaired platelet aggregation is not a unique characteristic of SARS-CoV-2 infection because reduced aggregation responses assessed using WBIA have previously been described with other viral infections [[48], [49], [50]] and sepsis [51]. This observation is corroborated by our finding that aggregation parameters below the reference range occurred in ∼40% of the SARS-CoV-2–negative patients (Figure 2), and more than half of those had infectious diseases other than COVID-19.

Second, reduced aggregation responses are not linked to previous platelet activation *in vivo* resulting in “exhausted platelets.” The expression of surface P-selectin (Figure 3) and activated GPIIb/IIIa (Figure 4) was negligibly low at the baseline in the patients with COVID-19, with no differences in comparison with that in the patient or healthy controls (P-selectin) or with even significantly lower expression levels compared with that in both the control groups (activated GPIIb/IIIa). Further, stimulation with various agonists *in vitro* induced normal surface expression of P-selectin in the SARS-CoV-2–positive patients. In contrast, we observed impaired GPIIb/IIIa activation in response to stimulation with the PAR1 agonist TRAP-6. However, a PAR-1–specific receptor defect is unlikely because TRAP-6–induced expression of P-selectin in the SARS-CoV-2–positive patients was not affected. Indeed, a quadrant analysis revealed that the percentage of P-selectin^+^/activated GPIIb/IIIa^−^ platelets upon TRAP-6 stimulation is increased by 2-fold in SARS-CoV-2–positive patients compared with that in healthy subjects (Supplementary Figure). This is in accordance with recent findings by Weiss et al. [52], suggesting uncoupling between α-granule secretion and GPIIb/IIIa activation in COVID-19. Impaired GPIIb/IIIa activation in patients with COVID-19 upon agonist stimulation *in vitro* has been described before [21,38,41] and was associated with a fatal outcome [27]. However, the underlying mechanism is still unclear, and the present study cannot provide an explanation for this observation.

Our study has the following limitations. The number of patients with COVID-19 was small, and no control for confounding was undertaken. This is primarily because of the prospective study design enrolling all patients with suspected SARS-CoV-2 infection prior to the PCR test result. Unexpectedly, only approximately one-third of patients with COVID-19–like symptoms turned out to be SARS-CoV-2 positive upon PCR testing or were tested positive before inclusion. We cannot exclude the fact that the timing of the positive test result may have affected the outcome. In addition, we performed flow cytometry analyses only on a subset of patients because the study was initially designed to examine only platelet aggregation. Because of lacking information on race or ethnicity, we could not control for sociocultural influences. Another limitation is that samples were collected once at the time of admission, which does not allow longitudinal analyses.

In summary, in patients at early stages of COVID-19, the proportion of circulating PNCs is increased. These heterotypic cellular conjugates can be associated with upregulation of neutrophil adhesion molecules and elevated responsiveness of neutrophils upon stimulation *in vitro*. In contrast, increased activation of circulating platelets or increased responsiveness *in vitro* is not detectable at this disease stage. We, therefore, conclude that primary platelet activation is not a mandatory condition for the formation of PNCs during COVID-19. Furthermore, enhanced generation of PNCs appears to be unique to SARS-CoV-2 infection because this phenomenon is not present in patient controls with COVID-19–like symptoms. Because of the limitations of our study, the conclusions drawn remain preliminary. Larger studies are needed to examine whether pharmacologic targeting of increased neutrophil activation can be an effective and safe strategy for early intervention in patients with COVID-19.
