# Supplementary material for: Early SARS-CoV-2 infection: Platelet-neutrophil complexes and platelet function
Source: Res Pract Thromb Haemost. 2022 Dec 23;7(1):100025. doi: 10.1016/j.rpth.2022.100025 (PMC9783187; doi:10.1016/j.rpth.2022.100025)
Supplement: Supplementary material [file mmc1.pdf]

Table S1

Patient	Days tested positive before inclusion	Days with symptoms before inclusion
1	0	2
2	8*	10
3	9	10
4	28*	35
5	0	3
6	0	4
7	5*	1
8	30*	0
9	9*	9
10	1*	7
11	0	2
12	0	10
13	6	8
14	6	8
15	0	3
16	2	8
17	7*	10
18	0	3
19	5*	10
20	8*	11

Table S1: Timing of initial positive PCR test and symptoms. The number of days tested positive before inclusion and the number of days with symptoms before inclusion are provided for each patient. Seven patients were tested positive for the first time at inclusion (0 days), whereas 13 patients were tested positive between day 1 and 30 prior to inclusion. The asterisks indicate that patients were retested positive on the day of inclusion (n=9). One patient was tested negative at inclusion (patient 3), and three patients were not tested again (patients 13, 14, 16). Except for one patient without any symptoms before the day of inclusion (0 days), all patients had symptoms beginning on various days prior to inclusion (range 1-35 days).

Figure S1

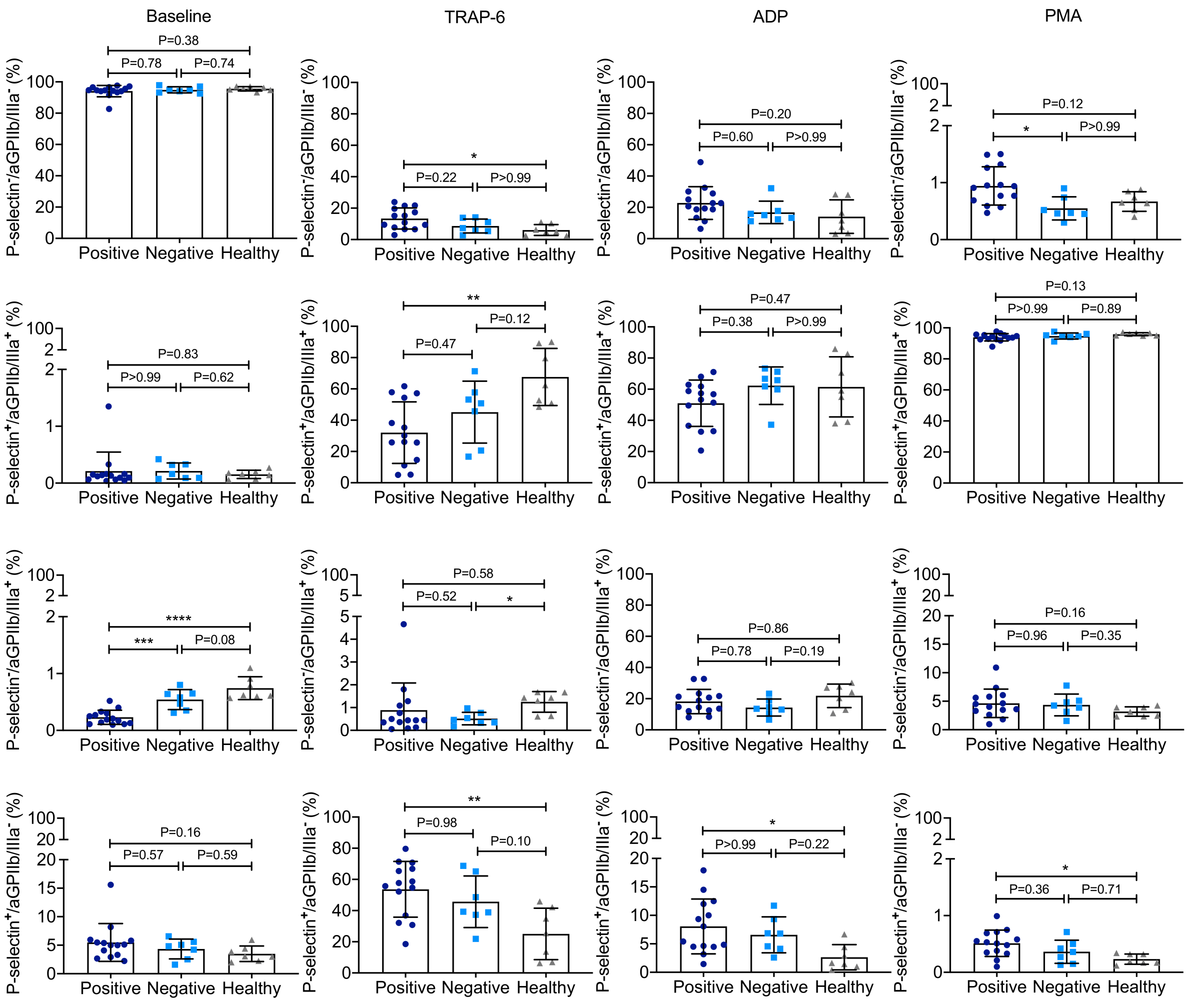

Figure S1: Impaired GPIIb/IIIa activation is uncoupled from α-granule secretion in SARS-CoV-2 positive patients. P-selectin and activated GPIIb/IIIa (aGPIIb/IIIa determined by PAC-1 binding) on the platelets surface was assessed in hirudinized whole blood (1:6 diluted) from SARS-CoV-2 positive patients (dark blue circles, n=14), SARS-CoV-2 negative patients (light blue squares, n=7), and healthy volunteers (grey triangles, n=7) after incubation with PBS (baseline), thrombin receptor activating peptide 6 (TRAP-6, 20 μM), adenosine diphosphate (ADP, 20 μM), or phorbol 12-myristate 13-acetate (PMA, 100 nM) for 15 min using flow cytometry. The percentage of double negative (P-selectin$^-$/aGPIIb/IIIa$^-$, first row), double

positive (P-selectin$^+$/aGPIIb/IIIa$^+$, second row), aGPIIb/IIIa single positive (P-selectin$^-$/aGPIIb/IIIa$^+$, third row) or P-selectin single positive (P-selectin$^+$/aGPIIb/IIIa$^-$, fourth row) platelets was determined by quadrant analysis. Data presented are individual values and mean ± standard deviation of each group. Data were analyzed using one-way ANOVA with Bonferroni's multiple comparisons test or Welch ANOVA with Games-Howell's multiple comparisons test. *P < 0.05; **P < 0.01; ***P < 0.001; ****P < 0.0001
